# Extended Survival after Complete Pathological Response in Metastatic Pancreatic Ductal Adenocarcinoma Following Induction Chemotherapy, Chemoradiotherapy, and a Novel Immunotherapy Agent, IMM-101

**DOI:** 10.7759/cureus.435

**Published:** 2015-12-26

**Authors:** Mafalda Costa Neves, Alex Giakoustidis, Gordon Stamp, Andy Gaya, Satvinder Mudan

**Affiliations:** 1 Department of HPB Surgery, The Royal Marsden NHS Foundation Trust; 2 Section of Investigative Medicine, Division of Diabetes, Endocrinology & Metabolism, Faculty of Medicine, Imperial College; 3 London Oncology Clinic, Guy's and St. Thomas' NHS Foundation Trust; 4 Department of Academic Surgery, The Royal Marsden NHS Foundation Trust; 5 Department of Surgery and Cancer, Imperial College London

**Keywords:** pancreatic adenocarcinoma, immunotherapy, chemotherapy, neoadjuvant chemoradiation, imm-101

## Abstract

Pancreatic ductal adenocarcinoma (PDAC) has an extremely poor prognosis. Median survival for metastatic patients is six to nine months and survivors beyond one year are exceptional. Pancreatic cancer is resistant to conventional chemotherapy and is often diagnosed at advanced stages. However, immunotherapy is a rapidly advancing new treatment modality, which shows promise in many solid tumor types.​

We present a patient with metastatic pancreatic cancer who underwent a synchronous resection of the primary tumour (pancreatoduodenectomy) and metastatic site (left hepatectomy) after multimodality neoadjuvant treatment with gemcitabine, nab-paclitaxel, and immunotherapy backbone with IMM-101 (an intradermally applied immunomodulator), as well as consolidation chemoradiation. Pathology of the specimens showed a complete response in both sites of the disease. The patient remains alive four years from the initial diagnosis and continues on maintenance immunotherapy.

This exceptional response to initial chemo-immunotherapy was followed by a novel and off-protocol approach of low-dose capecitabine and IMM-101 as a maintenance strategy. The survival benefit and sustained performance status could set this as a new paradigm for the treatment of oligometastatic pancreatic cancer following response to systemic therapy and immunotherapy.​

## Introduction

Pancreatic ductal adenocarcinoma has a characteristically poor prognosis with a ratio of diagnosis and cancer-specific mortality approaching unity. This is a globally distributed disease showing a significant resistance to conventional chemotherapeutics [1].

The majority of patients have metastatic or locally advanced disease at presentation and are not considered to be surgical candidates. Of the ~ 10% of patients eligible for resection, ~ 30% will expect to have positive (R1, R2) resection margins. Clinical trial data suggest a small survival benefit from adjuvant chemotherapy, but any advantage from radiotherapy remains undetermined [2]. The use of powerful chemotherapy regimens in the neoadjuvant setting for ‘borderline resectable’ cases has lifted resection rates, but as yet, a translation into improved overall survival rates has not been clearly demonstrated [3]. The survival benefit of surgery to the primary in the presence of oligometastatic disease still remains unproven.

By contrast, in colorectal carcinoma, for example, the demonstration of tumour response to effective chemotherapy has rendered the presence of controllable liver metastases no longer a contraindication to surgery with a curative intent. We believe that with recent advances in systemic therapy and a greater understanding of tumour biology, the role of the immune system and, in particular, the stromal response to cancer allows us to reconsider the role of surgery in selected patients with locally advanced and oligometastatic disease of the pancreas [4].

Our goal is to illustrate our recent experience deploying multiple modalities of therapy in Stage 4 pancreatic cancer combining systemic cytotoxic chemotherapy, radiation, and immunotherapy to achieve a complete pathological response, as proven by successful surgical resection of the primary and metastatic sites. This exceptional response to initial chemo-immunotherapy was followed by an off-protocol maintenance strategy with prolongation of overall survival and sustained performance status.

## Case presentation

**Figure 1:**
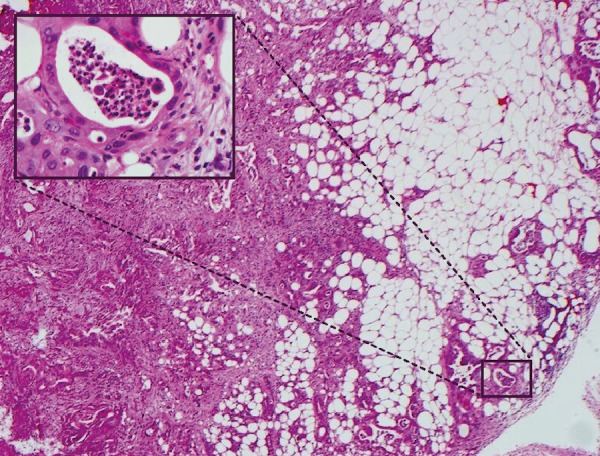
Duodenal pre-treatment biopsy The patient’s duodenal pre-treatment biopsy demonstrating a typical poorly differentiated ductal adenocarcinoma with distorted glandular structures embedded in the desmoplastic stroma. Detail of pleomorphic non-polarised ductal epithelial cells forming glandular lumina containing inflammatory cells, mostly neutrophils, is seen.​

A 72-year-old female, non-smoker, with an Eastern Cooperative Oncology Group Performance Status (ECOG PS) of 1 presented in January 2012 with jaundice, epigastric pain, and weight loss. Computerised tomography (CT) showed a 4 cm mass in the head of the pancreas, and her CA19.9 was 6,271 U/mL. A metallic bile duct stent was successfully placed, and duodenal mucosal biopsies revealed invasion by poorly differentiated PDAC (Figure 1). Staging was completed by FDG-PET scan, endoscopic ultrasound, and laparoscopy. The final staging was T4N1M0, and the patient considered for surgery. A trial dissection showed a common hepatic artery and coeliac axis encasement so the patient was deemed inoperable. Restaging prior to the commencement of palliative chemotherapy showed two new masses in the left liver, not evident on the preoperative staging, that were confirmed as metastases on FDG-PET scan.

In March 2012, the patient was entered into a Phase II clinical trial randomizing inoperable pancreatic cancer patients to gemcitabine alone versus the addition of the immunomodulator IMM-101 (NCT01303172) and allocated to the active arm.

IMM-101 is a suspension of heat-killed whole cell *Mycobacterium obuense *(NCTC13365) administered by intradermal injection [5]. It acts as a systemic immunomodulator, which has effects on innate and adaptive immune responses and may have application across a variety of tumor types.

After three cycles of the gemcitabine and IMM-101 combination, the CA19.9 dropped from 11,075 to 7,879 U/mL. A CT scan showed a partial response in the dimensions of both the primary and liver sites, according to RECIST criteria. After the sixth cycle, a CT scan showed disease progression in both sites with CA19.9 rising to 13,097 U/mL. However, she maintained an excellent quality of life and PS. After one month and with a CA19.9 of 38,000 U/mL, nab-paclitaxel was added to the gemcitabine and IMM101 combination. The patient demonstrated an excellent and sustained partial response for the following six cycles. The CA19.9 fell to 78 U/mL and the liver showed a complete radiological response, but the vascular encasement persisted. She was then offered consolidation chemoradiation two weeks after her last cycle, to a dose of 59.4 Gy in 33 fractions with concomitant capecitabine and IMM-101. Toxicity during radiation therapy consisted of Grade 2 fatigue, Grade 1 nausea, Grade 1 dyspepsia, loss of appetite, and weight loss of 4 kg. Following completion of radiotherapy, capecitabine and IMM-101 were continued as maintenance therapy.

Four months after chemoradiotherapy, an FDG-PET scan showed no avidity in either the primary tumour or the liver.

In view of her exceptional response and following discussion in a multidisciplinary meeting, it was agreed to re-attempt resection. In November 2013, twenty-two months after her initial diagnosis and six weeks after completion of chemoradiation, the patient underwent pylorus-preserving pancreatoduodenectomy with portal vein resection, left hepatectomy, and coeliac and retroperitoneal nodal dissection.

**Figure 2:**
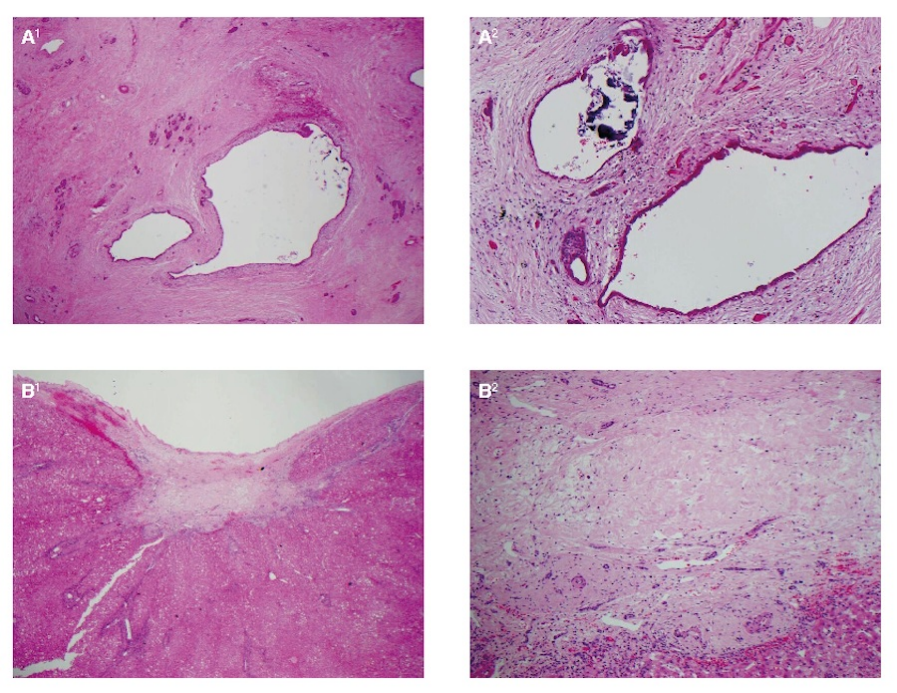
Post-treatment specimens A1 – Pancreatic resection specimen demonstrating atrophic pancreatic parenchyma in which there are atrophic acini and scattered islets but no malignant elements; A2 – The parenchyma contains residual benign ducts with islets embedded in fibrovascular scarring reaction. Some of the ducts contain dystrophic calcific aggregates; B1 – Low power view of the liver resection demonstrates a subcapsular dense collagen scar with no glandular elements; B2 – Examination at higher power confirms there is dense collagen with no residual carcinoma.

Intraoperatively, the primary tumour had regressed from the coeliac axis and skeletonisation of the common hepatic artery was possible. Scars were noted at the site of the metastases, but no mass was discernible on high-resolution intraoperative ultrasound scan. Histopathological analysis of the specimens showed a complete pathological response in both the primary site and liver (Figure 2). There was extensive fibrosis coupled with acinar atrophy extending right into the wall of the common bile duct. There were also extensive radiotherapy-associated changes with marked arterial intimal fibroplasia and microvascular obliteration. Additionally, the main pancreatic duct and its major branches exhibited active lymphohistiocytic infiltration with a predominance of lymphocytes - pathological stage ypT0 N0 (0/11) M0 V0 R0. Microsatellite instability was not part of the histopathological workup as, thus far, genetic heterogeneity has not shown a good correlation with the intrinsic immune response.

**Figure 3:**
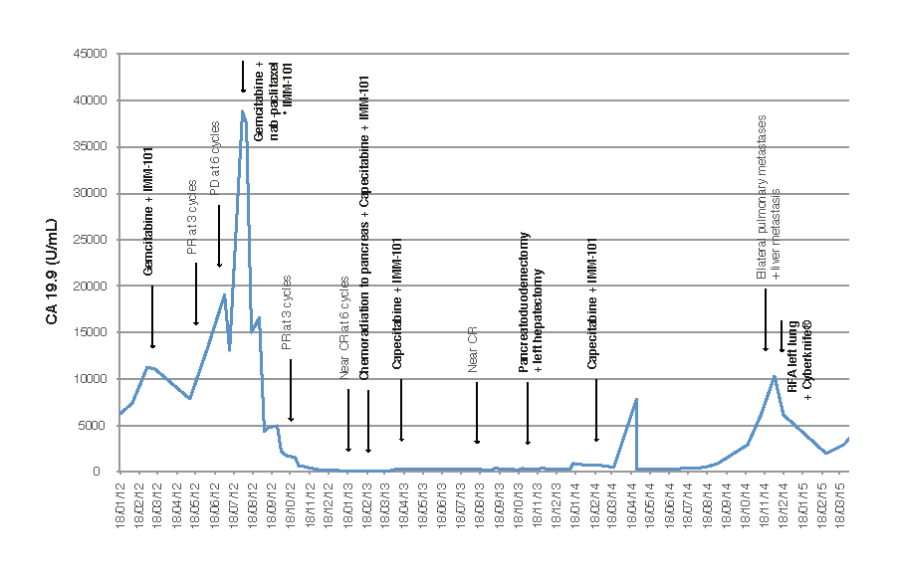
Treatment timeline according to the CA19.9 levels PR: partial response; PD: progressive disease; CR: complete response; RFA: radiofrequency ablation.

Postoperatively, the patient continued to receive IMM-101 and restarted capecitabine ten weeks after the operation. At twelve months from surgery, a CT scan showed bilateral small volume lung metastases and a further solitary lesion in the liver. She subsequently received radiofrequency ablation to both lungs, CyberKnife^®^ stereotactic radiation to the liver, and restarted gemcitabine + nab-paclitaxel in August 2015 following the development of a malignant pleural effusion. The treatment timeline according to the CA19.9 levels is shown in Figure 3. She maintains a good quality of life and has sustained the ECOG PS of 1 almost four years from diagnosis of her metastatic PDAC. 

Informed patient consent was obtained prior to her treatment. No identifying patient information is contained within this report. Written consent was also obtained from the patient for the publication of this case report and accompanying images.

## Discussion

We present a patient with metastatic pancreatic cancer who underwent synchronous resection of the primary tumour (pancreatoduodenectomy) and metastatic site (left hepatectomy) after multimodality treatment with gemcitabine, nab-paclitaxel, IMM-101, and consolidation chemoradiation. This resulted in a complete pathological response and prolonged overall survival and, to our knowledge, a first of its kind published in the literature with the use of immunotherapy.

Since the introduction of gemcitabine in 1997, further progress in therapy in the advanced/metastatic setting has been extremely low. Various Phase III studies have evaluated different gemcitabine-based regimens as a first line therapy but failed to show a consistent and significant improvement in survival in either the adjuvant or palliative setting. More recently, the Molecular Profiling-based Assignment of Cancer Therapy (MPACT) trial showed a significant survival benefit in patients with metastatic pancreatic cancer treated first-line with gemcitabine, plus nab-paclitaxel, an agent active in tumour stroma, versus gemcitabine alone [1]. This combination improved progression-free survival from 3.7 months to 5.5 months and OS from 6.7 months to 8.5 months compared to gemcitabine alone. At 12 months, the survival rate was 35% with the combination versus 22% in monotherapy, translating into an absolute increase of 7% in survival. Moreover, survival at two years doubled, increasing from 4% to 9%. The FOLFIRINOX regimen, whilst more toxic, also demonstrates a significant survival benefit in metastatic disease [6].

Immune dysregulation is a key feature of cancer, which includes impaired cell-mediated immunity and Th2 bias [5]. Similar functional changes are manifested in chronic psychological stress [7]. Furthermore, there is growing evidence that the phenotypic behavior of a tumour is not only defined by the epithelial component but also by the tumour microenvironment. Galon, et al. have shown that the analysis of a specific type of intratumoural immune response was surpassing the TNM classification in multivariate analysis as a better predictor of prognosis and response to therapy and suggested that the precise analysis of the tumour microenvironment is essential to optimal patient management [8]. They have proposed the ‘immunoscore’ based on the ratio of lymphocyte populations (CD3/CD45RO, CD3/CD8 or CD8/CD45RO) to quantify the *in situ* immune infiltrate and hope to validate and integrate it in the future as a new component of the TNM staging system (TNM-I).

IMM-101 is a systemic immunomodulator containing heat-killed *Mycobacterium obuense* that is injected intradermally. It has successfully completed a Phase I study in melanoma demonstrating its safety with patients showing a dose-dependent local immune response [5].

Our patient was randomized to the active arm of the Immune Modulation And Gemcitabine Evaluation-1 (IMAGE-1) Phase II clinical trial evaluating the combination of IMM-101 and gemcitabine as a first-line treatment of metastatic pancreatic cancer. Updated results of this trial in patients with poor PS showed that IMM-101 was associated with improved survival at 12 months to 24% versus 11.5% in patients receiving gemcitabine alone. It also showed that, at 18 months, this survival was amplified to 18.3% for IMM-101 treated patients compared to 2.3% in the control group [9].

Initially, our patient demonstrated a partial response; however, she progressed after six cycles and nab-paclitaxel was added to the gemcitabine and IMM-101. After six months, she showed a sustained partial response on imaging and a marked tumour marker response. In view of the suggested alteration in tumour behavior, she received consolidation chemoradiation, continuing with IMM-101, and was operated on 18 months after starting treatment, in keeping with our neoadjuvant/downsizing pathway for locally advanced primary cancer. Most exceptionally, the specimens showed a complete response both in the primary and metastatic sites in the liver. This exceptional response to initial chemo-immunotherapy was followed by a novel and off-protocol approach of low-dose capecitabine and IMM-101 as a maintenance strategy. Remarkably, she has remained disease-free for twelve months and is still alive four years after her initial diagnosis. This survival benefit and sustained performance status could set this as a new paradigm for the treatment of oligometastatic pancreatic cancer following response to systemic therapy and immunotherapy [10].

## Conclusions

This case illustrates several rare, if not unique, clinical observations:

1. A complete pathological regression of both primary pancreatic and metastatic disease, a very rare event.

2. The efficacy of gemcitabine with or without nab-paclitaxel on an immunotherapy backbone of IMM101 reflects the promising results of the IMAGE-1 trial.

3. The subsequent disease progression and sustained response to sequential anticancer challenges are in keeping with our current understanding of the mechanisms of effect from cancer-directed immunotherapy.

4. The maintenance of performance status in the face of prolonged chemotherapy exposure, permitted by the use of an unconventional low dose rather than the maximum tolerated dosing schedule.

5. The clinical behavior illustrates the characteristic tail to survival curves found in trial data of immunotherapy studies in cancer.
